# Supplementary material for: Chirality and energy transfer amplified circularly polarized luminescence in composite nanohelix
Source: Nat Commun. 2017 Jun 6;8:15727. doi: 10.1038/ncomms15727 (PMC5467208; doi:10.1038/ncomms15727)
Supplement: Supplementary Information — Supplementary Figures, Supplementary Methods and Supplementary References [file ncomms15727-s1.pdf]

# Supplementary Methods

**Synthesis of CNSB-Ac.** The ester form CNSB-Es was synthesized according to the reported procedures.[1] Briefly, the mixture of methyl 4-(cyanomethyl) benzoate (1.75 g, 1 mmol) and methyl terephthaladehydate (1.64 g, 1 mmol) in tertbutyl alcohol (30 mL) was stirred at 50 °C. Potassium tert-butoxide (2.31 g, 2.05 mmol) powder was dropped into the mixture and stirred for 2 hours. The resulting precipitate was used for next step without purification. The hydrolysis of CNSB-Es was carried on the general procedure. To a suspension of 1.65 g (0.5 mmol) DCS-Es in 75 mL 1:1 mixture of THF/MeOH, 15 mL of a 2 M KOH aqueous solution was added. The mixture was allowed to reflux for 3 h. THF was removed under reduced pressure and the resulting suspension was diluted with water. The precipitate formed by acidification with aqueous HCl (2 M) was collected by filtration, washed several times with water yielding 1.17 g (80%) of a white solid. The obtained CNSB-Ac was washed several times by water, ethanol and acetone because of the pretty poor solubility. It was used for next step without further purification.

**Synthesis of L-1/D-1.** LGAm and DGAm were synthesized according to previous reported methods.[2] LGAm/DGAm (2.40 g, 0.5 mmol) and CNSB-Ac (0.70 g, 0.24 mmol) were dispersed into a solution of THF/$CHCl_3$ (100 mL, 2:1, v/v). Then EDC•HCl (1.15 g, 0.6 mmol) and HOBt (0.81 g, 0.6 mmol) were added. The reaction mixture was stirred at 50 °C for 12 h. After removal of solvents, the remained solids were dissolved in THF (20 mL) and then poured into 1% $Na_2CO_3$ aqueous solution (500 mL). The collected precipitate was purified by column chromatography ($CHCl_3$) over silica gel to yield a yellow powder (0.85 g, yield: 26%). **L-1**: $^1$H NMR (300 MHz, $CDCl_3$): δ = 8.19-8.17 (d, 2H), 8.02-7.99 (m, 6H), 7.78-7.75 (d, 2H), 7.62 (s, 1H), 6.91 (s, 2H), 5.79 (s, 2H), 4.63-4.59 (m, 2H), 3.32-3.25 (m, 8H), 2.67-2.59 (m, 2H), 2.41-2.33 (m, 2H), 2.23-2.19 (m, 4H), 1.61-1.43 (m, 8H), 1.35-1.27 (m, 72H), 0.91-0.86 (m, 12H) MALDI-TOF-MS, (dithranol matrix): calculated for $C_{75}H_{125}N_7O_6$ 1220.0; found 1243 $[C_{75}H_{125}N_7O_6 + Na]^+$,1259 $[C_{75}H_{125}N_7O_6 + Ka]^+$. Elemental analysis: calculated for C, 73.79; H, 10.32; N, 8.03; found C, 74.01; H, 10.53; N, 7.98. **D-1**: $^1$H NMR (300 MHz, $CDCl_3$): δ = 8.19-8.17 (d, 2H), 8.02-7.99 (m, 6H), 7.78-7.75 (d, 2H), 7.63 (s, 1H), 6.88 (s, 2H), 5.82 (s, 2H), 4.63-4.59 (m, 2H), 3.34-3.28 (m, 8H), 2.67-2.59 (m, 2H), 2.41-2.33 (m, 2H), 2.26-2.18 (m, 4H), 1.61-1.43 (m, 8H),1.35-1.27 (m, 72H), 1.35-1.27 (m, 72H), 0.94-0.89 (m, 12H). MALDI-TOF-MS, (dithranol matrix): calculated for C75H125N7O6 1220.0; found 1220. Elemental analysis: calculated for C, 73.79; H, 10.32; N, 8.03; found C, 73.54; H, 10.21; N, 8.35.

# Supplementary Figures

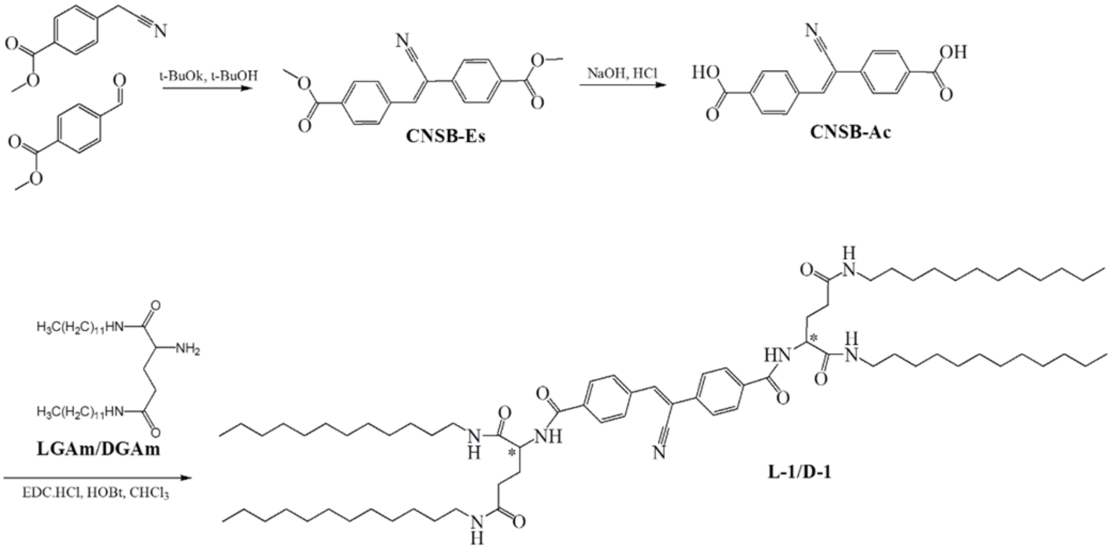


**Supplementary Figure 1.** Synthetic route of L-1 or D-1.

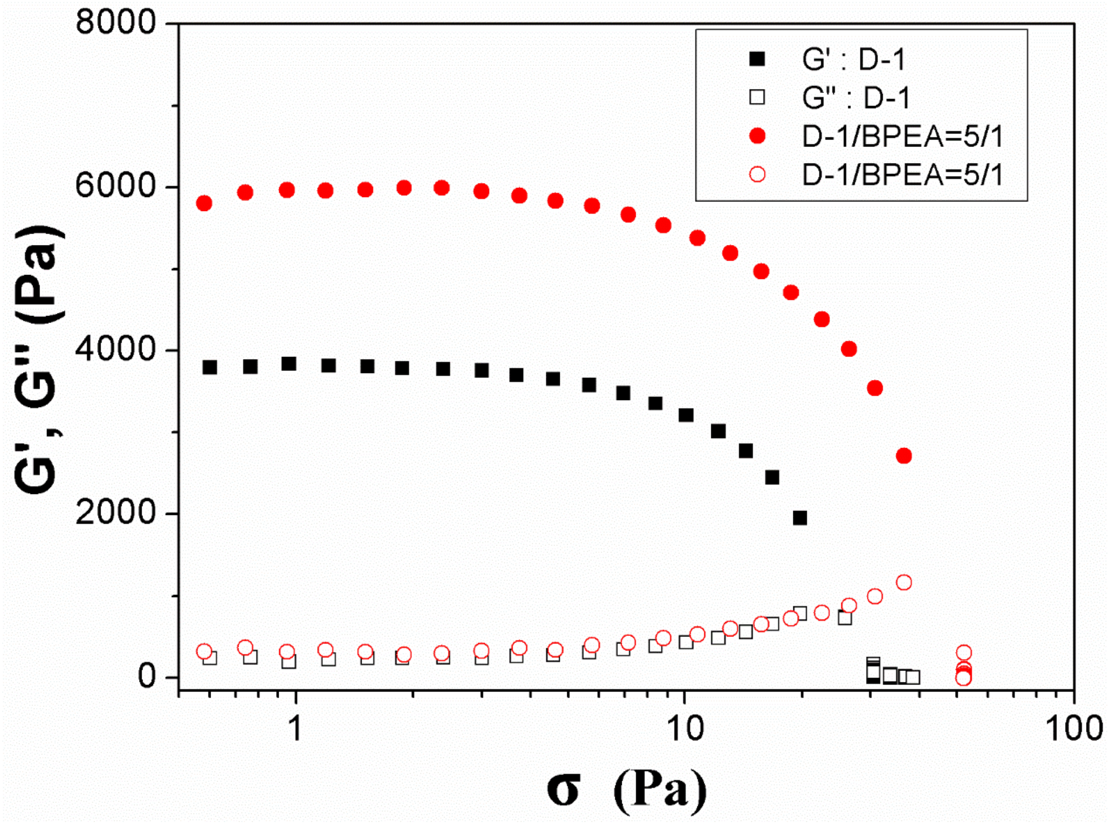


**Supplementary Figure 2.** Stress sweep rheology of the single-component D-1 gel ([D-1] = 2 mM) and two-components D-1/BPEA gel ([D-1] = 2 mM, [BPEA] = 0.1

mM). The measurement was conducted at constant 25 $^{o}$C and the measurement frequency was fixed as 0.1 Hz.

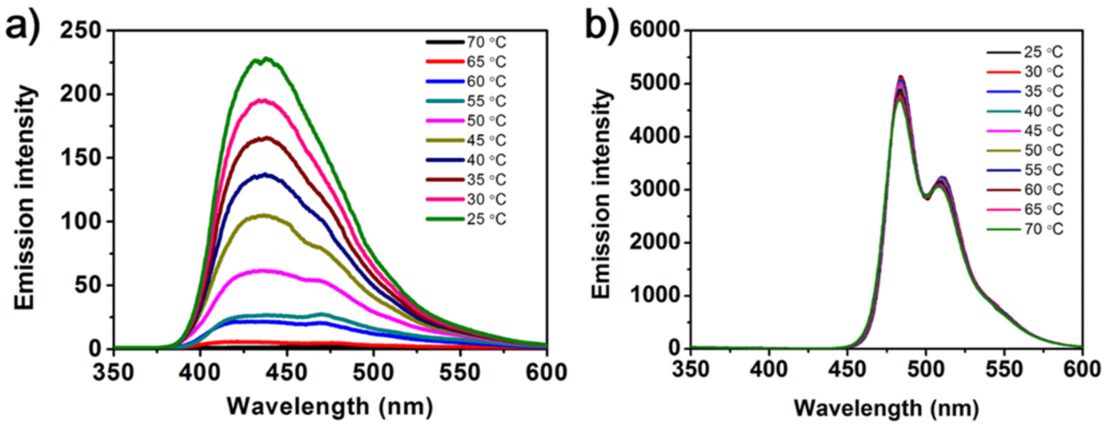


**Supplementary Figure 3.** Temperature dependent fluorescence spectra of (a) L-1 (2 mM) and (b) BPEA (0.4 mM) in DMSO/$H_2O$ (v/v = 9/1), $\lambda_{ex}$ = 320 nm. L-1 showed obvious assembly-induced emission enhancement. It can be calculated that the emission intensity of gel is around 100 times higher than the hot solution. Temperature dependent emission of BPEA in mixed solvent (0.4 mM) showed very slightly intensity change during the testing temperature which clearly indicates that there is no obvious aggregating behaviors in mixed solvent. This also confirms that BPEA also is an ideal energy acceptor.

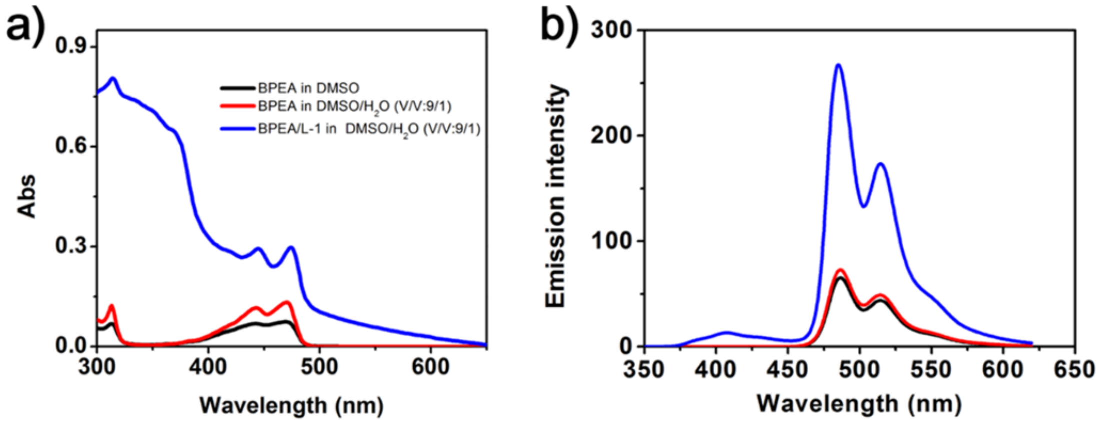


**Supplementary Figure 4.** UV-vis (a) and FL (b) spectra of BPEA in DMSO (black line), DMSO/$H_2O$ mixed solvent (red line, v/v = 9/1) and BPEA/L-1 co-gel in DMSO/$H_2O$ mixed solvent (blue line, v/v = 9/1, BPEA/L-1 = 1/5): [BPEA] = 0.4 mM, [L-1] = 2 mM, $\lambda_{ex}$ = 320 nm. Although the co-gel of BPEA/L-1 showed strong scattering in quartz cell, the obtained fluorescence spectrum exhibited around 4 times higher than the BPEA only in mixed solvent.

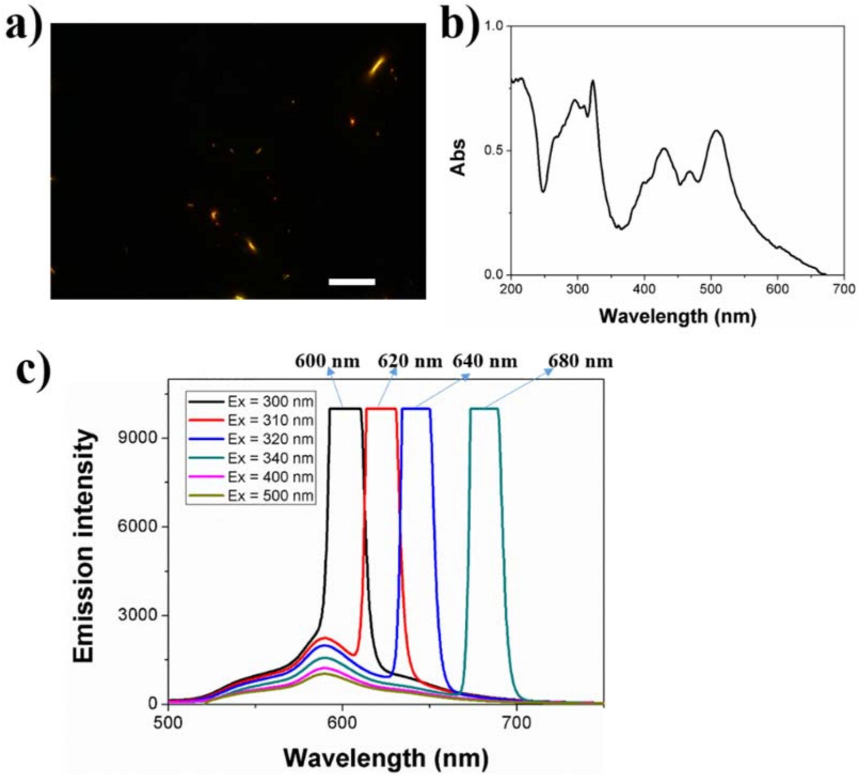


**Supplementary Figure 5.** (a) Fluorescent image of BPEA cast film and corresponding (b) UV-vis and (c) FL spectra. For the measurement of Fluorescent image of BPEA, the excitation light wavelength $\lambda_{ex}$ is 325 ~ 375 nm. BPEA solution in $CHCl_3$ was dropped on the surface of quarts plate and dried in air. For the measurement of emission spectrum of BPEA film, different excitation wavelength including 300 nm, 310 nm, 320 nm, 340 nm, 400 nm, 500 nm were used. The 600 nm, 620 nm, 640 nm, 680 nm emission peaks are attributed to the 1/2 fraction frequency fluorescence peaks. The emission slit, excitation slit and PMT voltage are 5 nm, 5 nm, 700 V. The scale bar for the fluorescent image is 10 μm.

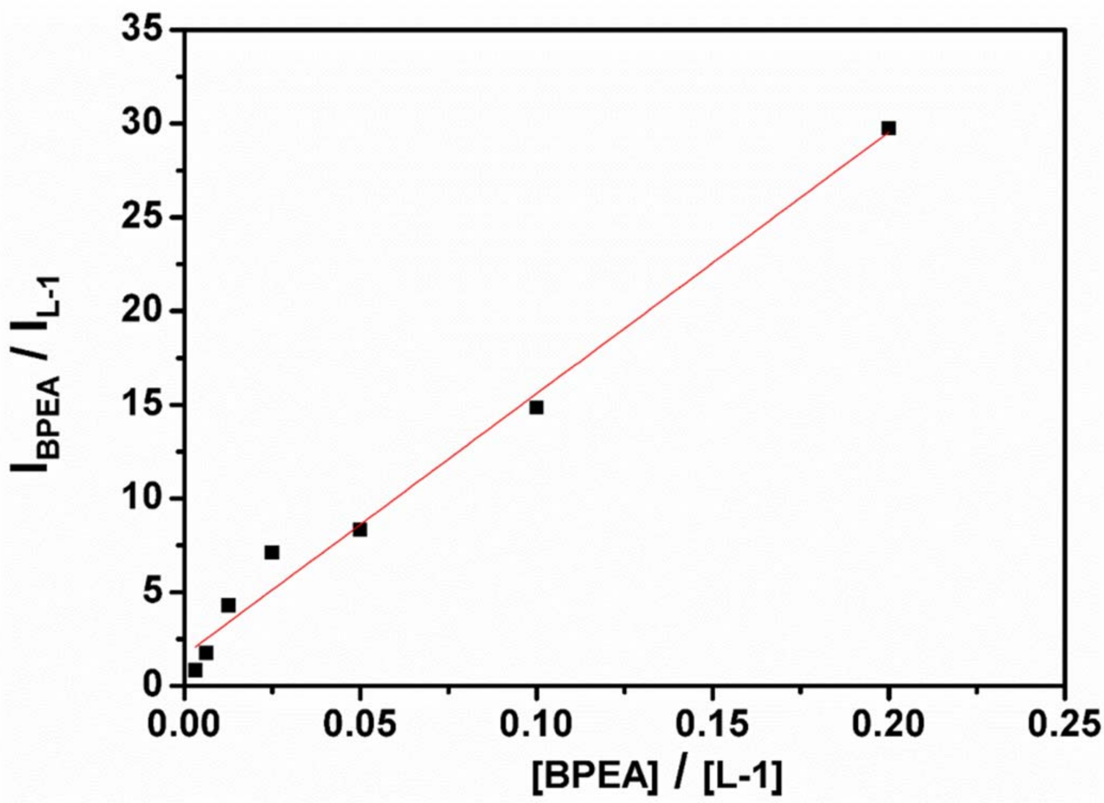


**Supplementary Figure 6.** Plot of relative fluorescence intensities of BPEA to L-1 against their molar ratio upon excitation at 320 nm. DMSO/H2O (v/v = 9/1), [L-1] = 2 mM, [BPEA] = 0.4 mM.

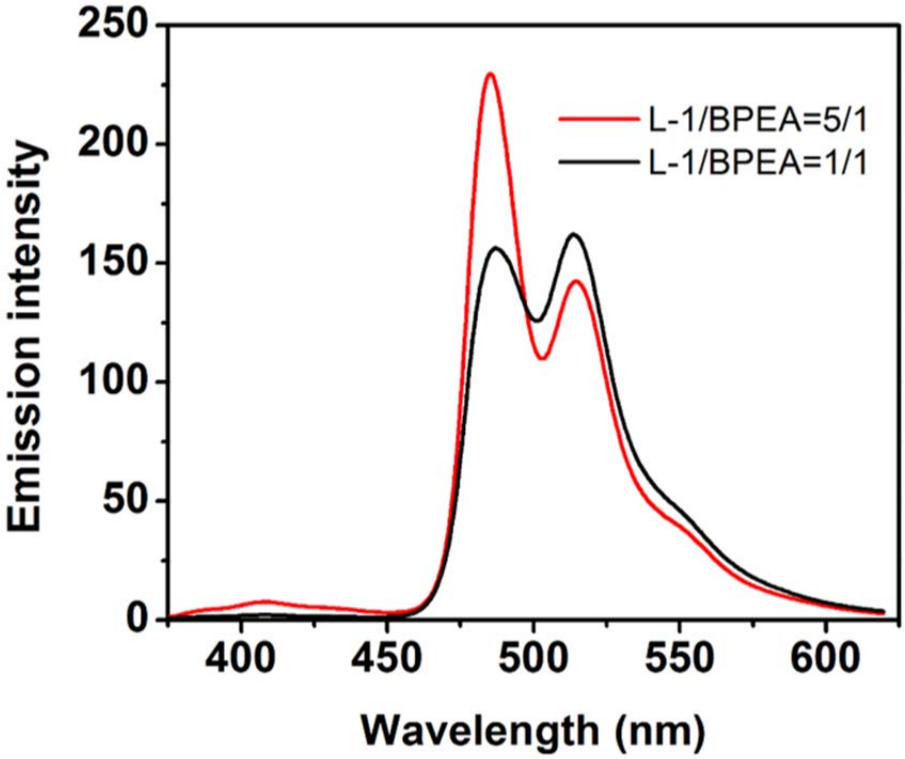


**Supplementary Figure 7.** Fluorescence spectra of L-1/BPEA mixture (black:

L-1/BPEA = 1/1, [BPEA] = 2 mM, [L-1] = 2 mM; red: L-1/BPEA = 5/1, [BPEA] = 0.4 mM, [L-1] = 2 mM) in DMSO/$H_2O$ (v/v = 9/1), $\lambda_{ex}$ = 320 nm. It is obvious that the FRET emission of BPEA showed quenching and broadening after the mixing ratio reaching up to 1/1.

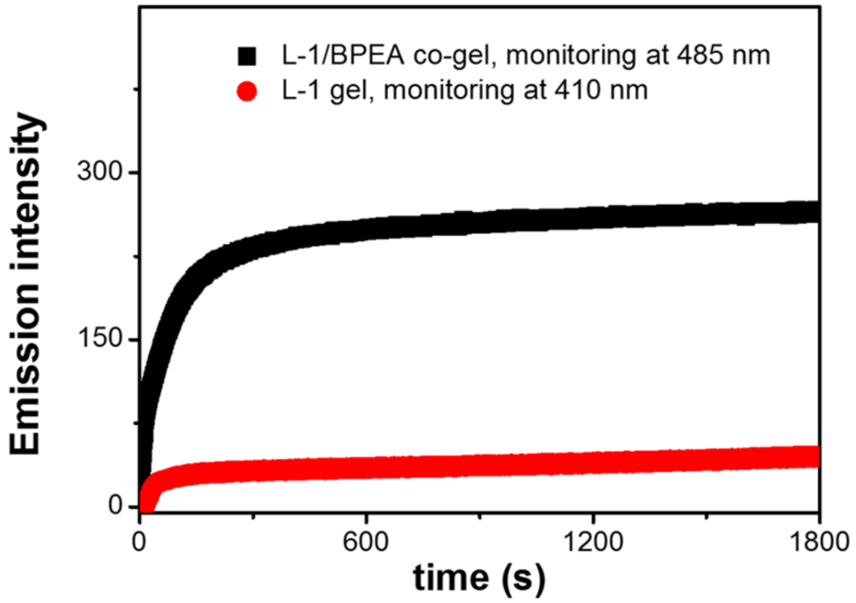


**Supplementary Figure 8.** Time-dependent fluorescence intensity of L-1 monitored at 410 nm and L-1/BPEA co-gel monitored at 485 nm.

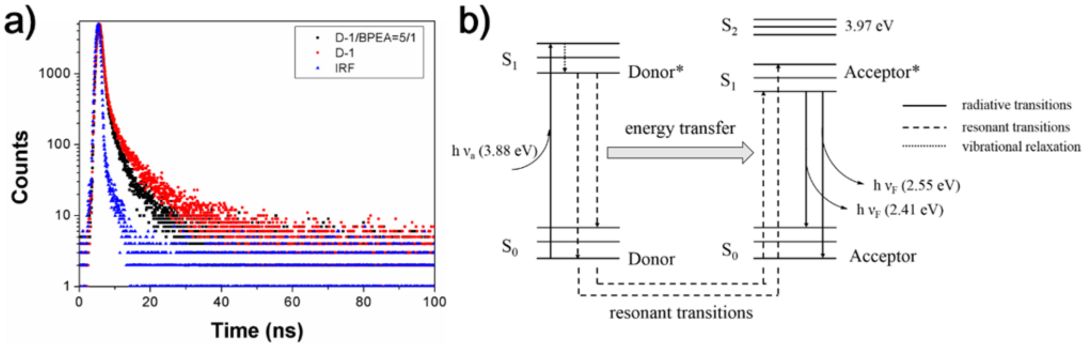


**Supplementary Figure 9.** a) Emission decay curves of the D-1 and D-1 / BPEA = 5/1 gels monitored at 450 nm. $\lambda_{ex}$ = 358.4 nm, IRF= instrument response function, [D-1] = 2mM, [BPEA] = 0.4 mM. b) The Jablonski energy diagram for the chiral donor and achiral acceptor. When absorbing 320 nm light, the chiral donor was excited to higher vibrational level of either $S_1$ or $S_2$, then rapidly relaxed to the lowest vibrational level of $S_1$. The energy of donor at $S_1$ state was transferred to acceptor at $S_0$ state through resonant transitions. The acceptor at $S_1$ excited state then decayed to different vibrational level of $S_0$ state with 487 nm and 514 nm fluorescence emission. The $S_0$-$S_1$ transition associated with 320 nm absorption for D-1 donor is expressed as 3.88 eV. The $S_0$-$S_2$ transition associated with 312 nm absorption for BPEA acceptor is expressed as 3.97 eV. The two emission bands located at 487 nm and 514 nm for

BPEA are expressed as 2.55 eV and 2.41 eV.

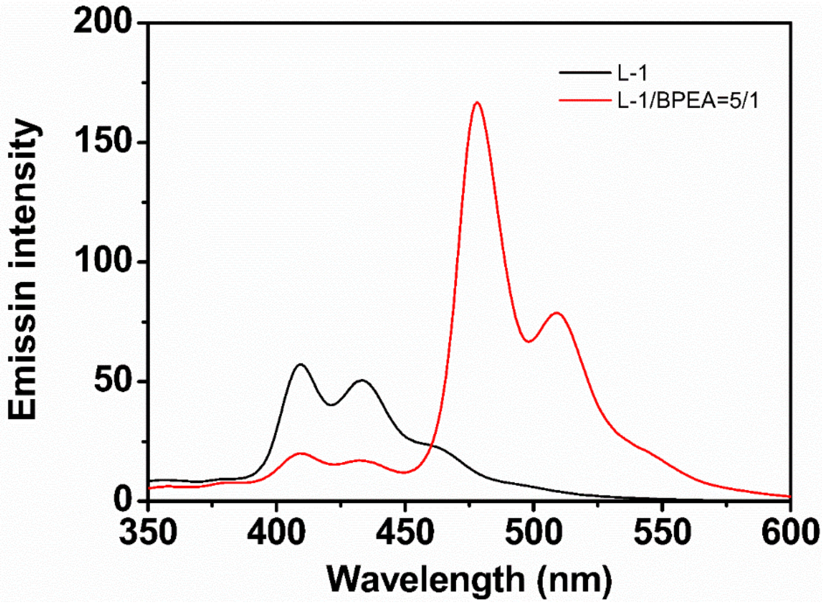


**Supplementary Figure 10**. The fluorescence intensity of L-1 and L-1/BPEA =5/1 in $CHCl_3$. [L-1]= 0.1 mM.

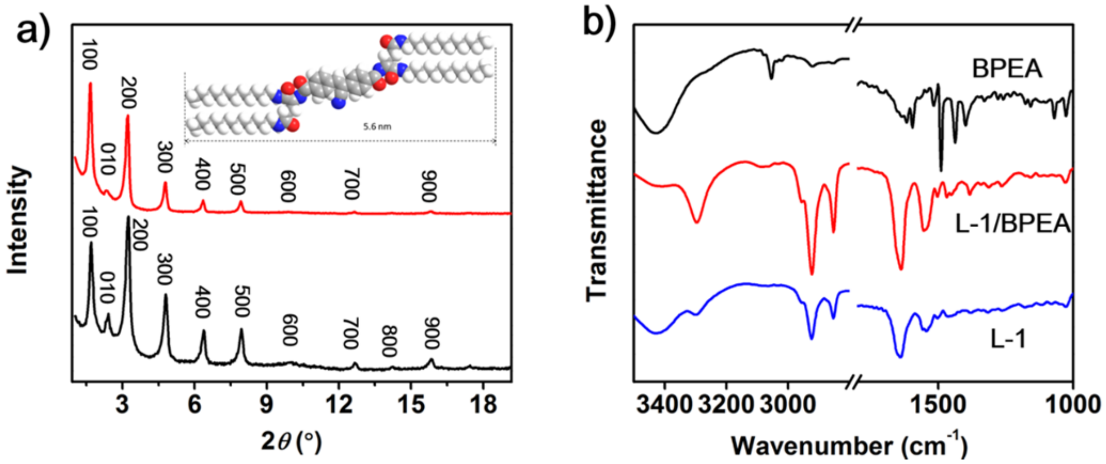


**Supplementary Figure 11.** (a) Power XRD of xerogel of L-1 (black line) and L-1/BPEA = 5/1(red line), (b) FTIR spectra of xerogel of L-1 (blue line), L-1/BPEA (red line) and BPEA (black line).

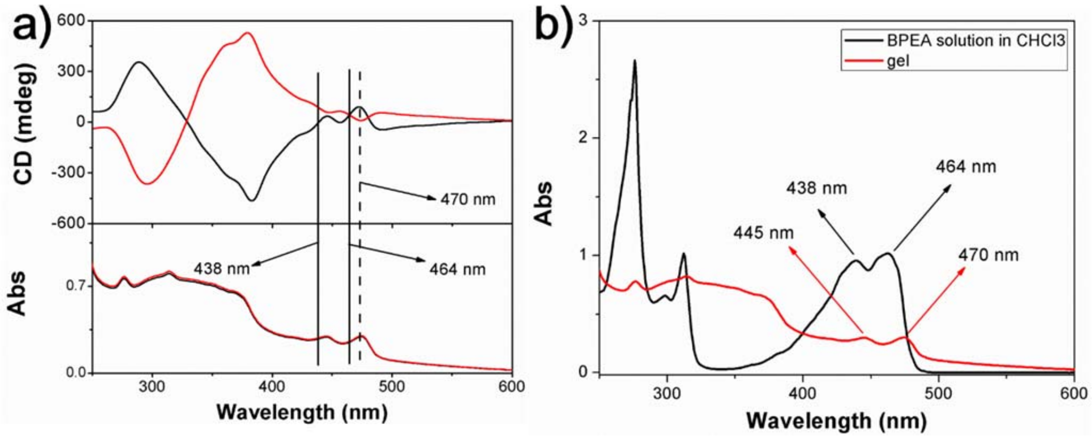


**Supplementary Figure 12.** a) CD and UV-vis spectra for L-1 (black line) and D-1 (red line) gels in DMSO/$H_2O$ (v/v = 9/1), [L-1] = [D-1] = 2 mM, [BPEA] = 0.4 mM. b) UV-vis spectra for D-1/BPEA gel in DMSO/$H_2O$ (red line ) and $3.17\times10^{-4}$ M BPEA in $CHCl_3$. 0.1 mm and 1mm cuvette were used in the UV-vis spectra measurement for gel and $CHCl_3$ solution respectively.

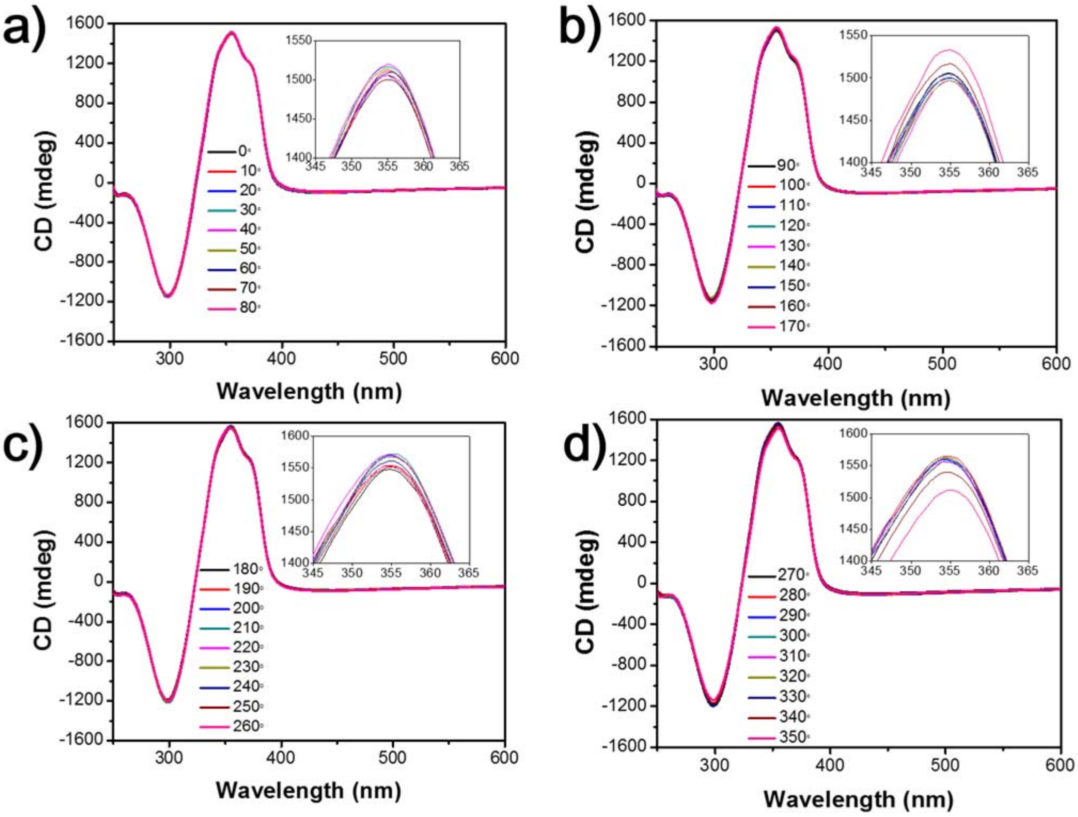


**Supplementary Figure 13.** 36 CD spectra of D-1 gel when rotating the sample about the optical axis in steps of 10°(a) 0°~ 80°, (b) 90°~ 170°, (c) 180°~ 260°, (d) 270°~ 350°. In order to ensure that the solvent does not evaporate during the measurement process, the sample was sealed in the 1mm cuvettes. At the same time, the concentration of D-1 was reduced to 0.8 mM for an appropriate UV-Vis absorption

intensity. When the rotation angle varied from 0° to 350° with a step of 10°, the 36 individual CD spectra showed different CD amplitude. This result suggests that there exists LD effect in the observed CD spectra.

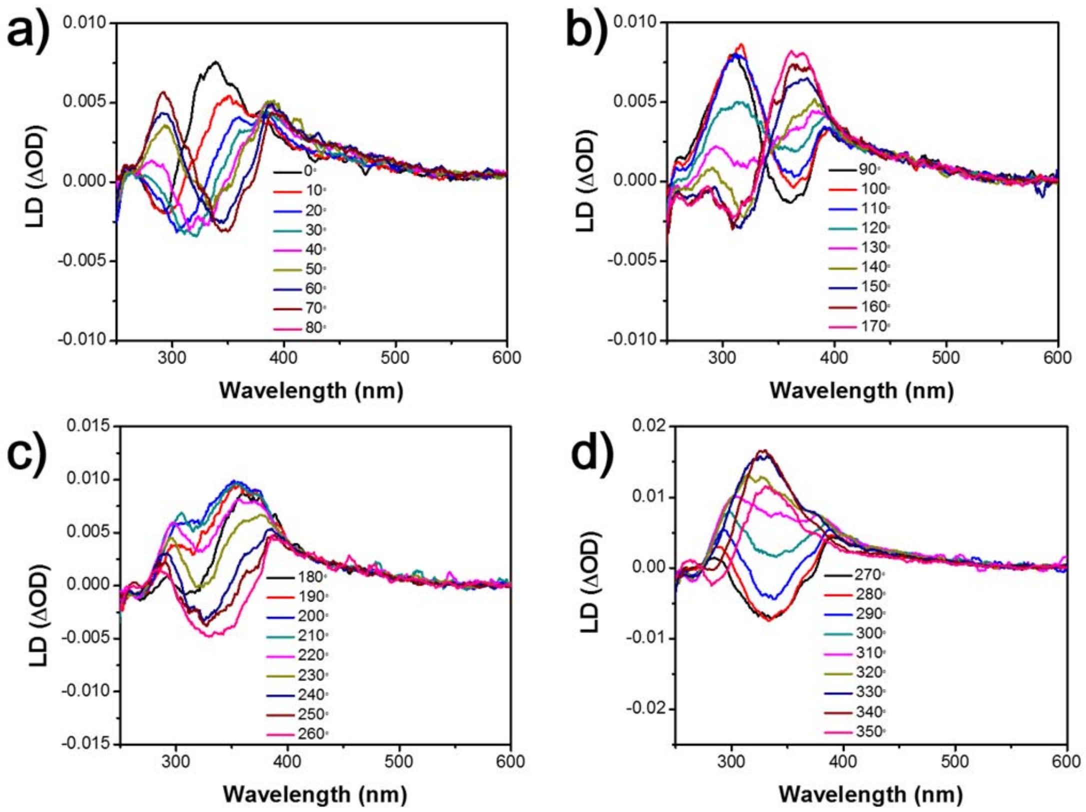


**Supplementary Figure 14.** 36 LD spectra of D-1 gel when rotating the sample about the optical axis in steps of 10° (a) 0°~ 80°, (b) 90°~ 170°, (c)180°~ 260°, (d) 270°~ 350°. The test condition was the same as that for CD measurement in Supplementary Figure 13. The obtained 36 LD spectra showed different LD curves, which indicated that there exists macroscopic anisotropic in the gel and thus results in LD artifacts.

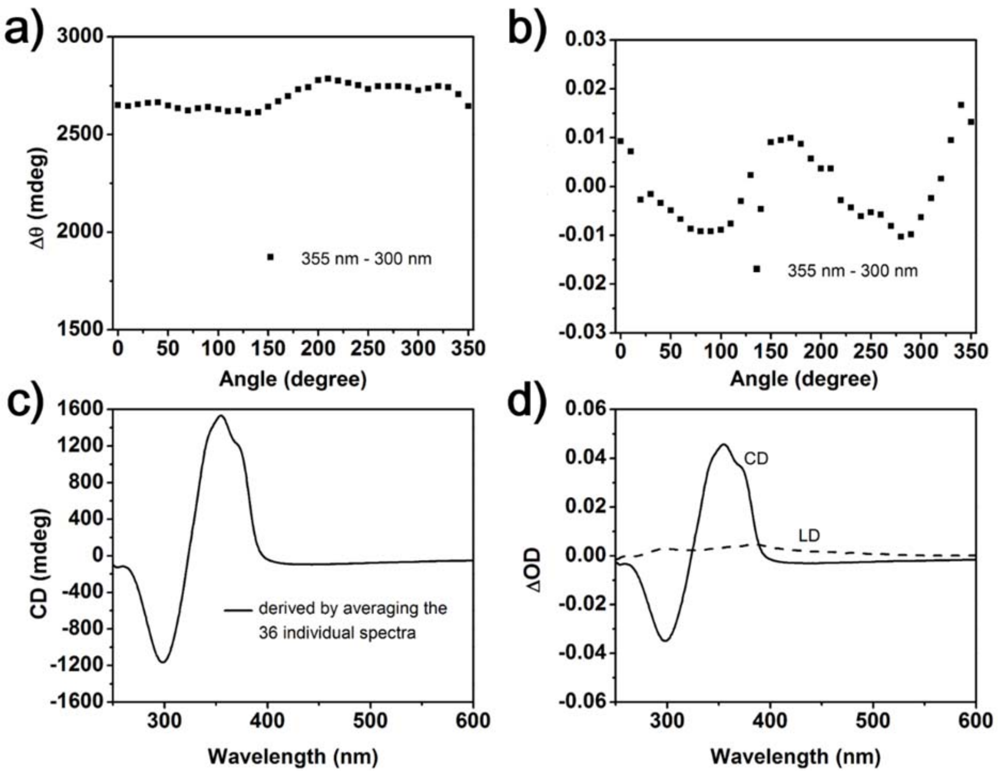


**Supplementary Figure 15.** (a) angle-dependent CD difference from the maximum value (355 nm) to the minimum value (300 nm) extracted from the 36 CD spectra in Supplementary Figure 13. (b) angle-dependent LD difference from the maximum value (355 nm) to the minimum value (300 nm) extracted from the 36 LD spectra in Supplementary Figure 14. (c) the mean spectrum of all 36 CD spectra.(d) the mean spectrum of all 36 CD spectra with the unit ΔOD for comparison(solid line) with the mean spectrum of all 36 LD spectra (dash line).

In Supplementary Figure 15a, the CD difference from the maximum value (355 nm) to the minimum value (300 nm) fluctuated around 2600 mdeg. This nonzero value suggested that the observed CD signal represents the authenticity of helical chirality in the gel system, though accompanied by some LD artifact. In fact, the true CD intensity can be obtained by averaging all of these 36 individual spectra as shown in Supplementary Figure 15c. In Supplementary Figure 15b, the angle dependence of LD amplitude adopted cosine function and was positioned around the zero line, which indicated that the LD effect can be eliminated by averaging all of the 36 individual LD spectra. We further use the semi-empirical equation (contamination of CD by LD = LD $\times 0.02 / CD_{observed}$) reported in literature to quantitatively estimate the contribution of LD to the CD spectra.[3] The contamination of CD by LD in the present case was estimated as about 0.26%.

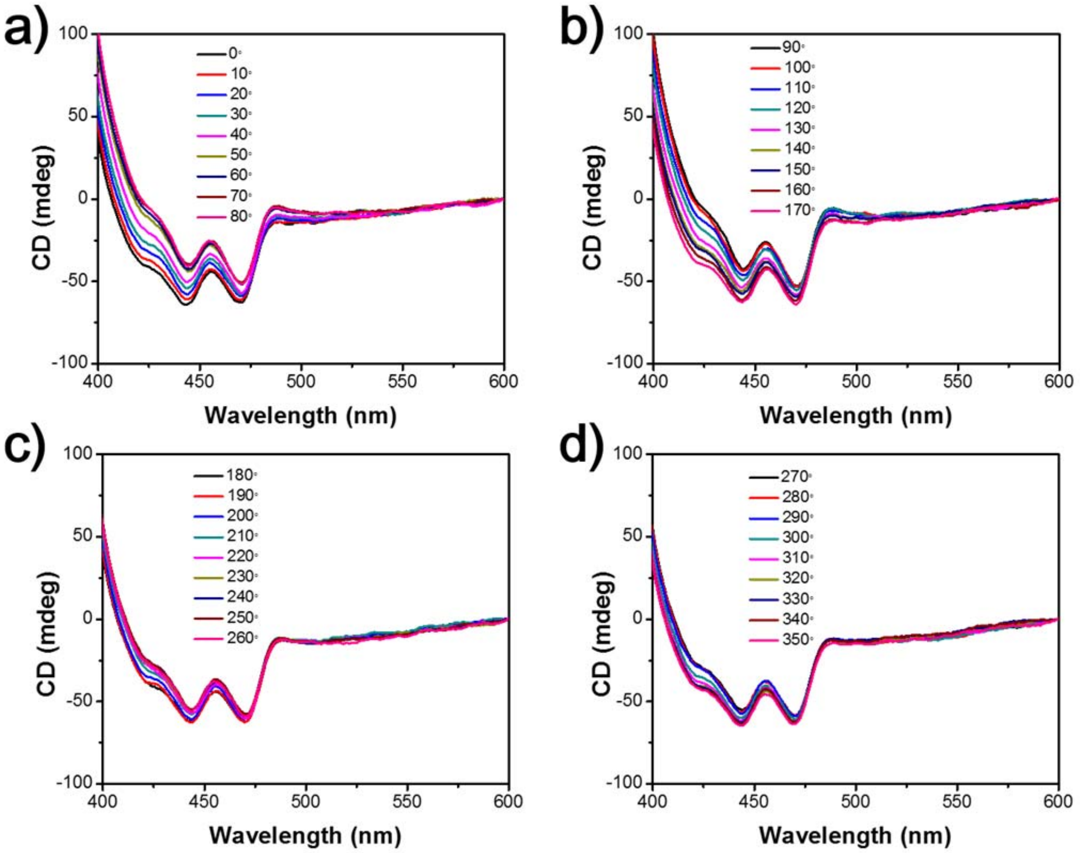


**Supplementary Figure 16.** 36 CD spectra of D-1/BPEA gel when rotating the sample about the optical axis in steps of 10°(a) 0°~ 80°,(b)90°~ 170°,(c)180°~ 260°, (d) 270°~ 350°. [BPEA] = 0.4 mM, [D-1] = 0.8 mM. For the clear observation of the induced CD signal for BPEA, the CD spectrum was only shown in the range of 400 nm - 600 nm.

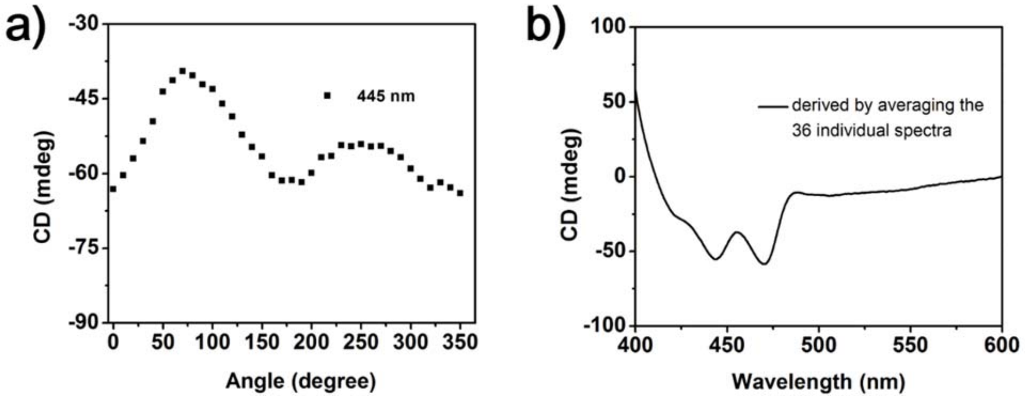


**Supplementary Figure 17.** (a) the dependence of CD signal of D-1/BPEA gel at 445 nm against the rotation angle in steps of 10°(b) the mean spectrum of all 36 CD spectra. The results indicated that the CD signal located in the absorption region of BPEA was truly from the induced helical chirality of the supramolecular system.

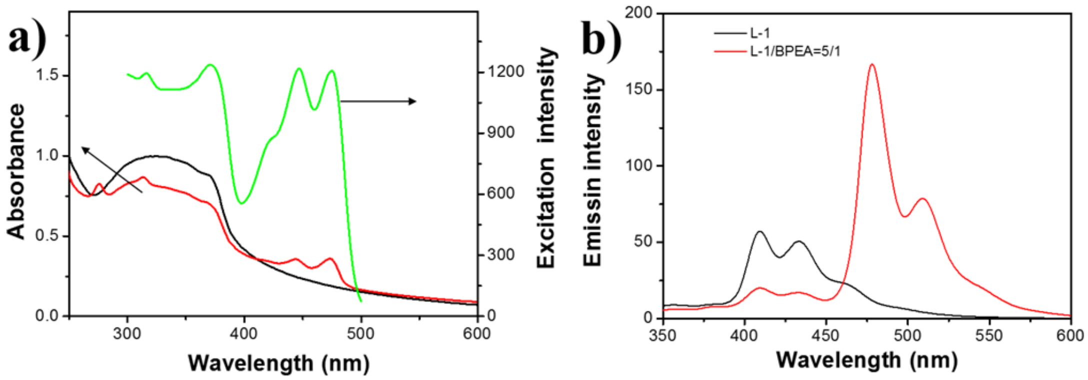


**Supplementary Figure 18.** (a) UV-Vis absorption spectra of L-1 gel (black line), L-1/BPEA co-gel (red line) and excitation spectra for L-1/BPEA monitored at 530 nm (green line). (b) Fluorescence spectra of L-1 ([L-1] = 0.4 mM) and L-1/BPEA ([L-1] = 2 μM, [BPEA] = 0.4 μM) mixture in $CHCl_3$.

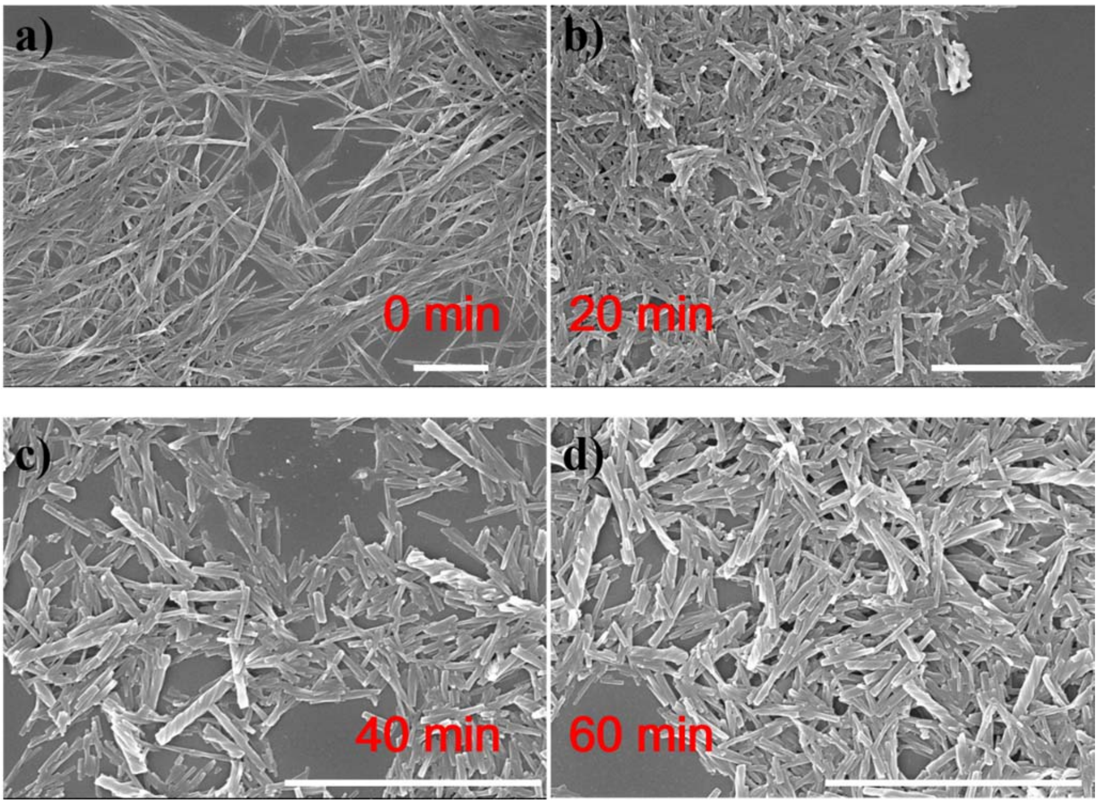


**Supplementary Figure 19.** SEM images of xerogel made from D-1/BPEA=5/1 with different ultrasonic time: a) 0 min, b) 20 min, c) 40 min, d) 60 min. The scale bars for are 5μm.

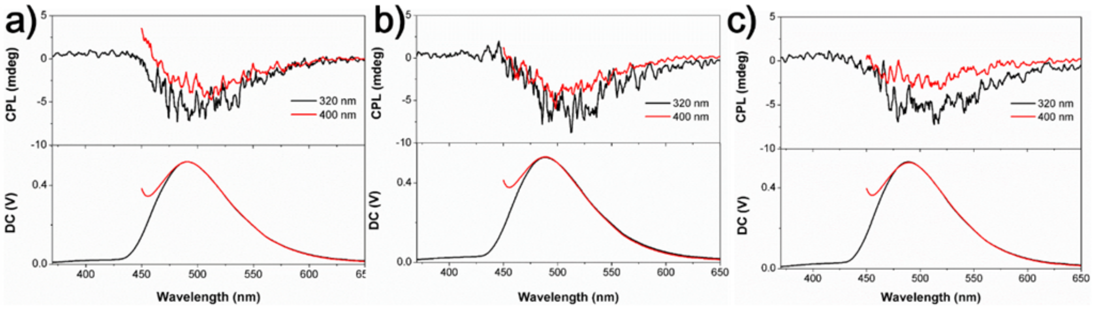


**Supplementary Figure 20.** CPL spectra of D-1/BPEA=5/1 gel with different ultrasonic time: a) 20 min, b) 40 min, c) 60 min. The samples were excited with 320 nm (black line) and 400 nm (red line) respectively. The ultrasonic treatment was conducted in KH5200E instrument and the power was kept constant at 200 W. The calculated CPL dissymmetry factors $g_{lum}$ at different ultrasonic time are as follow: a) $-5.9\times10^{-4}$ ($\lambda_{ex}$ = 320 nm), $-3.5\times10^{-4}$ ($\lambda_{ex}$ = 400 nm); b) $-8.6\times10^{-4}$ ($\lambda_{ex}$ = 320 nm), $-6\times10^{-4}$ ($\lambda_{ex}$ = 400 nm); c) $-7.4\times10^{-4}$ ($\lambda_{ex}$ = 320 nm), $-3\times10^{-4}$ ($\lambda_{ex}$ = 400 nm).

## Supplementary References


1 Yoon S. J. & Park S., Polymorphic and mechanochromic luminescence modulation in the highly emissive dicyanodistyrylbenzene crystal: secondary bonding interaction in molecular stacking assembly *J. Mater. Chem.* **21**, 8338-8346 (2011).

2 Li Y., Wang T. & Liu M., Gelating-induced supramolecular chirality of achiral porphyrins: chiroptical switch between achiral molecules and chiral assemblies. *Soft Matter*, **3**, 1312-1317 (2007)

3 Spitz C., Dähne S., Ouart A. & Abraham H.W., Proof of chirality of J-aggregates spontaneously and enantioselectively generated from achiral dyes. *J.Phy.Chem. B*, **104**, 8664-8669 (2000).
